# Evidence of tenofovir resistance in chronic hepatitis B virus (HBV) infection: An observational case series of South African adults

**DOI:** 10.1016/j.jcv.2020.104548

**Published:** 2020-08

**Authors:** Jolynne Mokaya, Tongai G Maponga, Anna L McNaughton, Marije Van Schalkwyk, Susan Hugo, Joshua B Singer, Vattipally B Sreenu, David Bonsall, Mariateresa de Cesare, Monique Andersson, Shiraaz Gabriel, Jantje Taljaard, Eleanor Barnes, Wolfgang Preiser, Christo Van Rensburg, Philippa C Matthews

**Affiliations:** a Nuffield Department of Medicine, University of Oxford, Medawar Building, South Parks Road, Oxford OX1 3SY, UK; b Division of Medical Virology, Stellenbosch University / National Health Laboratory Service Tygerberg, Cape Town, South Africa; c Division of Infectious Diseases, Department of Medicine, Stellenbosch University / Tygerberg Academic Hospital, Cape Town, South Africa; d MRC-University of Glasgow Centre for Virus Research, Bearsden Road, Glasgow, G61 1QH, UK; e Department of Infectious Diseases and Microbiology, Oxford University Hospitals NHS Foundation Trust, John Radcliffe Hospital, Headley Way, Oxford OX3 9DU, UK; f Big Data Institute, Old Road, Oxford OX3 7FZ, UK; g Wellcome Centre for Human Genetics, Roosevelt Drive, Oxford OX3 7BN, UK; h Division of Gastroenterology, Department of Medicine, Stellenbosch University / Tygerberg Academic Hospital, Cape Town, South Africa; i Department of Hepatology, Oxford University Hospitals NHS Foundation Trust, John Radcliffe Hospital, Headley Way, Oxford OX3 9DU, UK; j National Institutes of Health Research Health Informatics Collaborative, NIHR Oxford Biomedical Research Centre, John Radcliffe Hospital, Headley Way, Oxford OX3 9DU, UK

**Keywords:** HBV, HIV, Tenofovir, TDF, Resistance, RAMs, Africa, Nucleos(t)ide analogue, Therapy, Viraemia

## Abstract

**Introduction:**

Tenofovir disoproxil fumarate (TDF) is widely recommended for treatment of chronic hepatitis B virus (HBV) infection because it is safe, affordable and has a high genetic barrier to resistance. TDF resistance associated mutations (RAMs) have been reported, but data are limited, particularly for Africa. We set out to identify potential RAMs in individuals with detectable HBV viraemia on TDF treatment.

**Methods:**

We recruited adults with chronic HBV infection from Cape Town, South Africa, identifying individuals with a TDF resistance phenotype, defined as persistent HBV vireamia despite >12 months of TDF treatment. We sequenced HBV DNA using MiSeq Illumina with whole genome target enrichment, and sought potential TDF RAMs, based on a pre-defined list of polymorphisms.

**Results:**

Among 66 individuals with chronic HBV (genotypes A and D), three met our clinical definition for TDF resistance, of whom two were coinfected with HIV. In one participant, the consensus HBV sequence contained nine polymorphisms that have been described in association with TDF resistance. Significant treatment non-adherence in this individual was unlikely, as HIV RNA was suppressed. TDF RAMs were also present in HBV sequences from the other two participants, but other factors including treatment non-adherence may also have had a role in failure of HBV DNA suppression in these cases.

**Discussion:**

Our findings add to the evidence that RAMs in HBV reverse transcriptase may underpin a TDF resistant phenotype. This is the first time these RAMs have been reported from Africa in association with clinical evidence of TDF resistance.

## 1 Introduction

Tenofovir disoproxil fumarate (TDF) is a first line nucleoside analogue (NA) agent used to treat chronic hepatitis B virus (HBV) infection [1,2]. Although the genetic barrier to TDF resistance is high [1,2], recent reports suggest possible HBV resistance to TDF [3,4]. A review of potential TDF resistance associated mutations (RAMs) highlights significant gaps and limitations in the current literature, demonstrating the need for further investigation [5].

TDF is also used as antiretroviral therapy (ART) for HIV [6,7]. Thus, in settings where HIV is widespread, population exposure to TDF is high, raising the potential risk of selection of resistance in both HIV and HBV [7]. Monitoring HIV viraemia in coinfected patients receiving TDF-based regimens provides insights about treatment compliance, as HIV suppression is a surrogate for adherence to therapy.

Improved insight into the prevalence and clinical significance of HBV RAMs is essential; selection and dissemination of TDF resistant HBV would limit the success of antiviral therapy for individual patients, and may undermine efforts to curb transmission in pursuit of elimination targets [[8], [9], [10]]. We identified patients in whom concerns about possible drug resistance were raised on account of persistent HBV viraemia despite TDF therapy. Sequencing HBV confirmed the presence of mutations that have previously been described in association with TDF resistance.

## 2 Methods

### 2.1 Clinical cohort

**Fig. 1:**
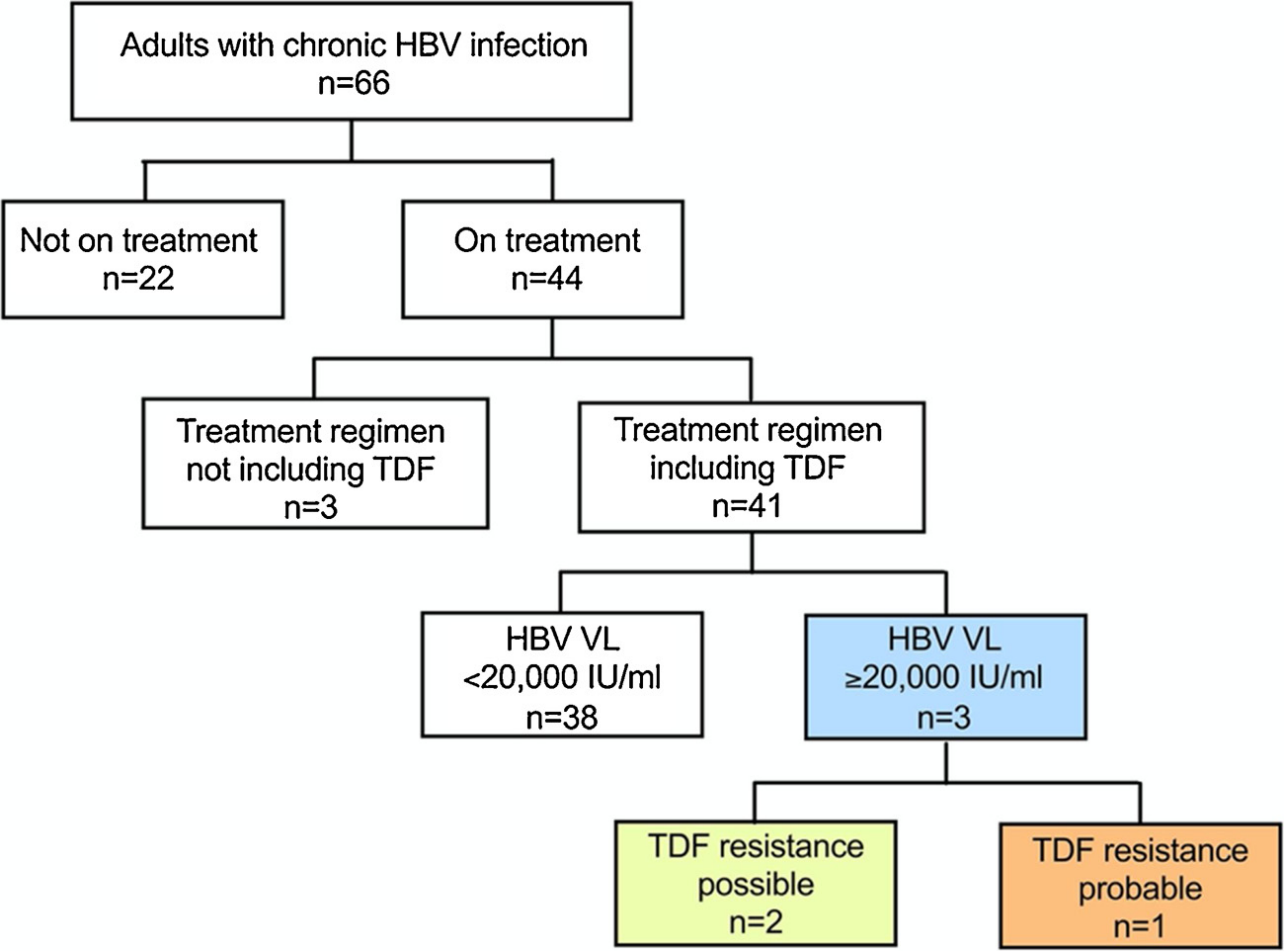
Selection of participants with chronic HBV infection for viral sequencing to identify tenofovir disoproxil fumarate (TDF) resistance associated mutations (RAMs). Adults attending clinical follow-up in Cape Town, South Africa, were enrolled over a period of one year. Three adults with persistent viraemia despite >12 months of TDF therapy are indicated in the blue box. Of these one had a phenotypic profile and HBV sequence data to support TDF resistance (orange box); two others had possible TDF resistance RAMs in HBV sequences but other factors including non-adherence to therapy may have contributed to persistent viraemia (yellow box). (For interpretation of the references to colour in this figure legend, the reader is referred to the web version of this article).

Adults were enrolled into a cross-sectional cohort of CHB at Tygerberg Hospital, Cape Town, South Africa (‘OxSAHep’ HBV study), as previously described [11]. All participants provided written informed consent. Ethics approval was provided by the University of Oxford Tropical Research Ethics Committee (ref. OXTREC 01–18) and Stellenbosch University Human Research Ethics Committee (HREC ref. N17/01/013). We identified patients with CHB in whom HBV viraemia was ≥20,000 IU/mL despite being on TDF treatment for >12 months (Fig. 1).

South African guidelines for HBV treatment recommend use of lamivudine (3TC), entecavir (ETV) or TDF [12,13]. In practice, TDF is most frequently used, as 3TC use is limited by drug resistance, and ETV is expensive and not widely available.

### 2.2 Deep sequencing and identification of RAMs

We extracted HBV DNA from plasma using a NucliSENS magnetic extraction system (BioMérieux), and used a published protocol to generate fully double stranded HBV genomes [14,15]. We purified DNA and prepared sequencing libraries followed by whole genome target-enrichment with custom-designed HBV probes. We sequenced the samples on an Illumina Mi-Seq using a v3 300bp paired end kit and analysed using Tanoti v1.3 [16] with genotyping and variant calling performed using the HBV-GLUE resource [17].We called TDF RAMs using a catalogue of 37 RAMs in HBV reverse transcriptase (RT) [5,7].

## 3 Results

### 3.1 Phenotypic evidence of TDF resistance

**Fig. 2:**
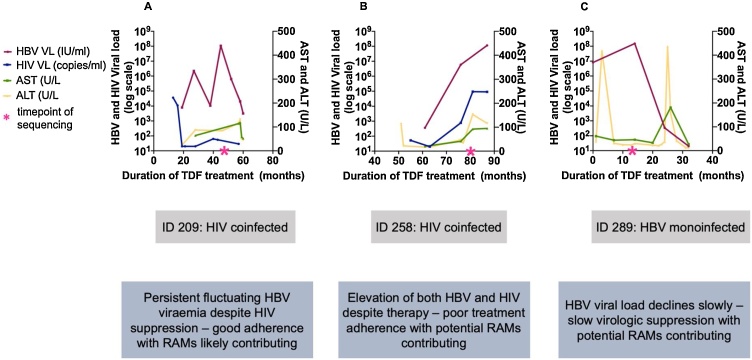
Longitudinal viral load and liver function test distribution of three adults with detectable HBV viraemia >20,000 IU/mL despite ≥12 months prescribed TDF therapy. Panels A-C plot laboratory parameters over time in three patients. The corresponding clinical data for each patient is shown below in panel D.

Among 66 adults with CHB, we identified three with HBV DNA viral load (VL) ≥20,000 IU/mL, despite therapy with TDF for ≥12 months (Figure 1), of whom two were co-infected with HIV. Both co-infected patients were treated with 3TC and Emtricitabine (FTC) in addition to TDF. In patient 209, HIV RNA suppression provides evidence for treatment adherence (Figure 2A Figure 2A ), increasing the probability that HBV drug resistance is genuinely present. In contrast, in patient 258, both HIV and HBV remained detectable, suggesting a possible incomplete adherence to therapy, with or without drug resistance in one or both viruses (Figure 2B Figure 2B).

A third case (ID 289) was HIV negative. HBV viral suppression occurred after a cumulative 30 months on treatment (Figure 2C Figure 2C). This individual also had concurrent large flares in liver enzymes (Figure 2 C Figure 2C).

### 3.2 Genomic evidence of TDF resistance

We derived full-length HBV genome sequences for all three patients, although with varying depth of coverage, identifying genotype-A in two cases and -D in the third (Table 1). The consensus sequences all contained RAMs that have been previously described in association with TDF resistance .

**Table 1:** Summary of patient characteristics and HBV sequence data generated from three South African adults. All patients had with persistent HBV viraemia ≥20,000 IU/mL despite >12 months prescription of TDF therapy.

Patient ID	209	258	289
Treatment	TDF + 3TC + FTC	TDF + 3TC + FTC	TDF
Age range at recruitment	41−50	41−50	61−70
HIV status	Positive	Positive	Negative
Elastography score (kPa)	6.9	5.1	9.1
HBV DNA viral load, log10 IU/mL at time of sequencing	8.03	6.77	8.18
HIV RNA viral load, log10 copies/mL at time of sequencing	1.74	4.97	N/A
Median coverage/site	4262	1601	14
HBV Genotype	A	D	A
Reverse transcriptase (RT) polymorphisms potentially associated with TDF resistance	Y9H	Y9H	Y9H
	L91I	F122L	L91I
	H126Y	S223A	H126Y
	R153W*	D263E	S223A
	V173L*, †	V278I	C256S
	L180M*, †	A317S	
	M204V*, †		
	S223A		
	C256S		

Underlined polymorphisms have been reported as RAMs, but occur frequently in the genotype identified in this patient (see Table 2).

* These RAMs have been identified based on a combination of both *in vivo* and *in vitro* data and are reported in ≥2 independent peer-reviewed sources, giving them the most stringent evidence base of all the polymorphisms listed [6].

† These RAMs cause HBV resistance to 3TC [8]. As patient 209 was on both TDF and 3TC, it is likely that exposure to 3TC resulted in the selection of RAMs V173L, L180M and M204V.

In HBV sequences from patient 209, we observed nine potential TDF RAMs. Among these, four polymorphisms (R153W, V173L, L180M, and M204V), have the strongest evidence base [5]. M204I/V and L180M are best recognised in association with 3TC resistance [19], but they may also be part of a combination of polymorphisms that underpins TDF resistance as there was no sustained viral suppression. In patients 258 and 289, six and five putative TDF RAMs were observed, respectively. At a deep sequence level, the RAMs we observed in all three patients were present in >95 % of HBV reads.

For each potential RAM identified in our HBV sequences we reviewed the prevalence of the variants in published HBV sequences (Table 2). In all three cases, a number of potential TDF RAMs actually represent the most common residue in this genotype [20]. Of note, for patient 289, four of five TDF RAMs are actually the most common residue at the site for genotype-A.

**Table 2:** Frequency of putative TDF RAMs in genotype A (n = 290) and D (n = 566) HBV sequences downloaded from Hepatitis B Virus Database (HBVdb), https://hbvdb.ibcp.fr/HBVdb/. 840 genotype A and 958 genotype D sequences were downloaded on the 20th of November 2018, and the datasets were filtered to remove highly similar sequences using a 99.5 % similarity threshold, leaving a total of 290 geno-A and 566 geno-D sequences.

Polymorphism in HBV RT	Frequency in genotype A sequences	Frequency in genotype D sequences
Y9H	286/ 290 (98.6 %)	556/566 (98.2 %)
L91I	290/290 (100 %)	41/566 (7.2 %)
F122Y/L	8/290 (2.8 %)	82/566 (14.5 %)
H/126Y	225/290 (77.6 %)	4/566 (0.7 %)
R153W	228/290 (78.6 %)	22/566 (3.9 %)
V173L	0/290 (0%)	4/566 (0.7 %)
L180M	7/290 (2.4 %)	13/566 (2.3 %)
M204I/V	7/290 (2.4 %)	25/566 (4.4 %)
S223A	289/290 (99.7 %)	563/566 (99.5 %)
C256S	279/290 (96.2 %)	65/566 (11.5 %)
D263E	1/290 (0.3 %)	111/566 (19.6 %)
V278I	16/290 (5.5 %)	55/566 (9.7 %)
A317S	4/290 (1.4 %)	531/566 (93.8 %)

## 4 Discussion

### 4.1 Summary of findings

We present the first evidence of potential TDF resistance in adults with CHB in Africa. The three patients presented reflect different phenotypes, with one case (ID 209, Figure 2A Figure 2A) representing particularly strong evidence for HBV resistance to TDF. The other two cases illustrate the difficulties in discriminating between drug resistance and incomplete adherence, and may also reflect the long timelines sometimes associated with viraemic suppression on TDF [21]. These explanations are not mutually exclusive, and may all have a role in incomplete virologic suppression.

### 4.2 Interpretation

Patient 209 was treated with both TDF and 3TC, making it likely that exposure to 3TC resulted in the development of RAMs V173L, L180M and M204V, possibly setting the scene for the subsequent development of cross-resistance to TDF. Although FTC is not part of the recommended treatment options for CHB, it is widely used for HIV and has some activity against HBV. Combination TDF and FTC has been shown to have better viral suppression compared to TDF monotherapy among treatment naïve individuals with CHB [22], suggesting these mutations may confer resistance to both drugs.

The precise contribution of these polymorphisms to resistance remains to be determined with certainty [5]. All putative RAMs identified in patient 289 occur frequently in genotype-A sequences, making it uncertain whether drug resistant polymorphisms have been selected *de novo* or are part of a founder sequence. This individual also had large flares in liver enzymes (Figure 2C Figure 2C) that may be evidence of a host immunological response in parallel with drug therapy accounting for the reduction in HBV DNA viraemia. Identifying putative RAMs, and ascertaining their significance to clinical phenotype, is more difficult when these polymorphisms represent the ‘wild type’ sequence in certain genotypes (as described for hepatitis C virus [24,25]). There is a pressing need for more clinical data combined with *in vitro* data from assays that can confirm a resistant phenotype.

### 4.3 Caveats and limitations

The data we present here are limited to only three individuals, with different clinical phenotypes (Fig. 2). In patient 289, despite a high viral load we achieved a limited sequencing coverage depth (Table 2). Incomplete treatment adherence can also result in virological breakthrough, and assessment of drug levels in plasma and/or PBMCs could provide more objective information [23], especially where HIV cannot be used as a surrogate measure of treatment compliance.

### 4.4 Summary

As TDF is widely used in Africa as a component of first line ART, it is possible that the selection of RAMs in HBV is more prevalent than previously reported [7,12]. Our findings add to the evidence that mutations in HBV RT can underpin a TDF resistant phenotype. This is the first time these RAMs have been reported from Africa supported by clinical evidence of TDF resistance. Further investigation is urgently needed to determine the prevalence and clinical significance of TDF RAMs, to investigate the role of combination therapy, and to support the development of new antiviral agents.

## Data availability

HBV sequence data are available at GenBank [accession numbers MT210032, MT210033, MT210034]

## Authors contribution statement

JM, TM and PM conceived the study. TM and PM acquired funding and ethics approval. TM, MvS, SH, JT, WP, CvR recruited patients and collected clinical data. JM, AM, DB and MdC generated sequence data. JM, TM, AM, JS *vs* and PM analysed the data. JM, AM and PM wrote the manuscript. All authors revised and approved the final manuscript.

## Financial support statement

This work was supported by the Leverhulme Mandela Rhodes Scholarship to JM, Wellcome Trust (grant number 110110Z/15/Z to PCM), the Medical Research Council UK to EB, the Oxford NIHR Biomedical Research Centre to EB. EB is an NIHR Senior Investigator. The views expressed in this article are those of the author and not necessarily those of the NHS, the NIHR, or the Department of Health.

## Declaration of Competing Interest

We have no conflicts of interest to declare.
